# Supplementary material for: Modulation of late Pleistocene ENSO strength by the tropical Pacific thermocline
Source: Nat Commun. 2020 Oct 23;11:5377. doi: 10.1038/s41467-020-19161-6 (PMC7584583; doi:10.1038/s41467-020-19161-6)
Supplement: Supplementary file 1 — Supplementary Information [file 41467_2020_19161_MOESM1_ESM.pdf]

Supplementary Information for Modulation of Late Pleistocene ENSO strength by the tropical Pacific thermocline

Rustic, Gerald T. [1,2 *], Polissar, Pratigya J. [2,3], Ravelo, Anna Christina [3], White, Sarah M.[4,5]

Author Affiliation:

1 – Department of Geology, School of Earth and Environment, 600 Whitney Ave, Rowan University, Glassboro, NJ USA 08028

2 – Lamont-Doherty Earth Observatory, Columbia University, 61 Route 9W, Palisades, NY USA 19604

3 – Ocean Sciences Department, University of California at Santa Cruz, Santa Cruz, CA USA 95064

4 – formerly at Earth and Planetary Sciences Department, University of California at Santa Cruz, Santa Cruz, CA USA 95064

5 – now at Department of Geography, University of California at Berkeley, Berkeley, CA 94720

*Corresponding author: rustic@rowan.edu

Supplementary Note 1: Individual foraminifera resampling and ENSO change

Individual foraminifera analysis has been used in previous studies to capture changes in ENSO in the past millennium ([1]), in the Holocene ([2], [3]), and at the LGM ([4]). Forward modeling of individual foraminifera distributions has demonstrated that individual foraminifera distributions can capture changes in mixed-layer variability, including ENSO change ([5]), and past ENSO change as recorded in sub-annual coral records has been captured in simulations of individual foraminifera sampling ([2], specifically Supplemental figure S5 in this study). Core-top calibration across the tropical Pacific has shown that SST distributions generated from Mg/Ca ratios in individual foraminifera accurately reflect local oceanic conditions and are sensitive to changes in ENSO variability ([6]). We demonstrate that the mixed-layer temperatures of our study site respond to changes in the seasonal cycle, ENSO frequency and ENSO amplitude. We further test the ability of individual foraminifera to capture changes in ENSO parameters by simulation of foraminifera selection via bootstrap resampling.

Forward modeling of CEP mixed-layer temperatures

We generated forward models to test the response of SST distributions to changes in the seasonal cycle, ENSO frequency, and ENSO amplitude using the 37m depth from the Carton 2.1.6 reanalysis data set (1958-2008) for the 17PC core site. We decomposed the monthly SST record into the seasonal cycle and a monthly anomaly time series. Alterations to the seasonal cycle were performed by changing the overall amplitude of the seasonal chance. ENSO amplitude change was simulated by altering the anomalies during recognized ENSO events. ENSO frequency change was simulated by adding (via duplication) or removing ENSO events (e.g., removing the anomalies). Random noise was applied to the resulting anomaly time series, and this was then recombined with seasonal cycle to regenerate a monthly temperature record.

Individual foraminifera selection.

To simulate the selection of individual foraminifera, we randomly selected 80 monthly temperatures from synthetic time series based on the SODA 2.1.6 mixed-layer temperature reanalysis data set. We altered the time series for both length (by appending copies of the time series) and for changes in ENSO amplitude and/or frequency to determine the parameters that ENSO (and more specifically, El Niño) change is most apparent. For each change in ENSO parameters, we performed the random selection of monthly temperature data 1000 times and applied random temperature uncertainty from a normal distribution with standard deviation equal to our analytical uncertainty (±0.47 °C) to simulate selection and analysis of individual foraminifera. We then calculated quantiles for each realization, then the mean value for every quantile from all realizations. We calculated a 90% confidence interval about that mean, and plotted the mean quantiles vs. the quantiles of the original, unmodified temperature series and the calculated confidence interval to test whether ENSO change is identifiable. We find that simulated sampling of individual foraminifera from an altered SST distribution is capable of capturing change in ENSO amplitude relative to the reference interval, and such changes appear in the tails of the distribution (**Supplementary Figure 1**). Changes in ENSO frequency does alter the distributions in the same fashion, with the same response, or with the same consistency, demonstrating that change in the tails of the distribution is likely the result of amplitude change (**Supplementary Figure 2**). We find that the number of individuals (60 vs 80) and the length of the synthetic time series the foraminifera are chosen from (50 vs 500 years) has a minor effect on the ability of the method to identify changes in the synthetic time series, and that denser sampling leads to higher chances of identification at lower ENSO change. Sampling rates comparable to our sample rates (80 specimens over 500 years) demonstrate that sampling analogous to individual foraminifera analysis will indicate decreases in ENSO amplitude of 19%

and increases in ENSO amplitude of 21% in over 90% of cases using the 98th quantile as a metric. Using the 96th quantile as a metric for ENSO change, ENSO amplitude changes of 21% and 25% for decrease and increase, respectively, are captured in 90% of simulations. For the 94th quantile, change in ENSO amplitude of 27% is captured in 90% of simulations.

Diagnostic features of quantiles

In these synthetic SST distributions, we find that the 94th, 96th and 98th quantiles respond strongly and linearly to changes in ENSO amplitude (**Supplementary Figure 3**). Significant changes in these quantiles may be identified with ENSO changes of ±10% in our SST simulations. In our simulations, the 98th quantile is the most sensitive to changes in ENSO amplitude, while changes in ENSO frequency or the seasonal cycle are not as sensitive at the 98th quantile. However, this quantile is subject to the highest frequency of false positive results, and thus analysis of additional quantiles and the total number of quantiles is necessary. Intervals with multiple quantiles showing deviation from the reference population have increasingly lowered frequencies of false positives, and we find that the mean of the 94th-98th quantile has a lower frequency of false positives than the 98th quantile while retaining diagnostic capability (**Supplementary Figure 3**).

Centennial-scale variability

We tested the impact of centennial-scale variability on the distribution of mixed-layer temperatures by generating multi-century time series beginning with 50-year modern monthly reanalysis temperature data and repeatedly appending the modern reanalysis data set. 2000 (**Supplementary Figure 4**) and 400 (**Supplementary Figure 5**) year time series were generated to bracket the length of time a given sediment interval in our cores may represent. In all simulations, random temperature anomalies from a normal distribution of mean of zero and standard deviation equal to initial reanalysis data set standard deviation were added. Centennial

scale variability was simulated by changing the properties of the resulting temperature data. We tested warming and cooling trends, sudden changes in average temperature, and random changes temperature in the appended data. To test trends, a trend of either + or - 0.05°C was added to each appended temperature set, resulting in an overall warming or cooling trend of ±0.1°C/ century. Changes in average mixed layer temperature were simulated by altering the mean temperature of one half of the appended intervals by ±0.5°C. The final test was the random addition of data with either a mean of +0.5°C, -0.5°C, or unchanged data. 80 months of the resulting temperature series were then randomly selected to simulate selection of individual foraminifera. Quantiles 0-100 at 2% intervals were calculated for these distributions. This process was repeated 1000 times and the median values for each quantile was calculated. These quantiles were then used for Q-Q analysis against the modern mixed-layer reanalysis data. We found that no simulations showed significant change in the distributions. The warming/cooling trends showed deviation, although not significant, from the 1:1 line. In the case of the 2000-year trends, an increase in mixed-layer temperature variability was observed as an increase in median absolute deviation (MAD). However, as this is unaccompanied by significant changes in the distributions, it is unlikely that our results represent this sort of trend. We conclude from these tests that non-ENSO centennial-scale variability is unlikely to manifest itself as a change in the extreme ends of the distributions that are used as indicators of ENSO strength.

Supplementary Note 2: Age Model

The age model for core 14MC1 is based on previous published radiocarbon dates ([2]). The age model for core ML1208-17PC used in this analysis is based on alignment of an existing *Globigerinoides ruber* $\delta^{18}O$ stratigraphy ([7]) with the LR04 benthic stack ([8]) using Analyseries 2.0.8 for Macintosh ([9]). We analyzed existing same-core benthic and planktic $\delta^{18}O$ from the

Western Pacific warm pool (ODP site 806, 0.19 °N, 159.22°E, 2520m) ([10], [11]) and Eastern Pacific warm pool (TR163-19, TR163-19, 2.25°N, 90.95°W, 2348m) ([11], [12]) to determine if benthic-planktic age offsets exist in the tropical Pacific Ocean. Benthic and planktic $\delta^{18}O$ records were normalized to the mean of their respective record, then plotted together as normalized $\delta^{18}O$ vs. depth to determine if the $\delta^{18}O$ records diverge at major marine isotope stage (MIS) boundaries, as identified visually in the records (Supplementary Figure 6). We find that the records show no appreciable or consistent offset between benthic and planktic records. Further, creation of new records using Analyseries at selected MIS boundary transition points resulted in planktic and benthic depth-age models that were nearly identical.

Existing age models for core 17PC have been based on an orbitally-tuned *Globigerinoides ruber* $\delta^{18}O$ stratigraphy, core-top radiocarbon dating, and Monte-Carlo based cross-correlation on sediments <150 ka ([7] ,[13]). For this study, we realigned the oxygen isotope stratigraphy to the LR04 benthic $\delta^{18}O$ stack ([8]) via multiple resampling and averaging of nine age control tie points to better characterize the major transitions observed in the record and to align the record in its entirety to the LR04 baseline. Resulting ages and previously published ages are listed in Supplementary Table **1**. Each control point was dated via manual alignment with the LR04 stack ten times using Analyseries, and the resulting age models were averaged to generate the final age model. Age control points are found in Supplementary Table 2. The maximum standard deviation among age control points is ±2.1 ky and the average standard deviation is ±0.72 ky. The resulting age model is within 2.1 ky of both previous age models within the last 150 ky. Sample ages for core 17PC were linearly interpolated between averaged age control points.

The Analyseries generated age model was compared to the additional age models, including those based on the LR04 benthic stack alignment (JLS)([7]), the Monte-Carlo cross correlation

methods ([13]), and the Hidden Markov Method Probstack generated $\delta^{18}O$ stratigraphy ([14]) (Supplementary Figure 7). Our ages, generated using Analyseries, are within 5ky of LR04 and Probstack predicted ages with the exception of MIS 8 where there is significant disagreement between the JLS and Probstack models. Our model falls between these and we believe more accurately aligns with state transitions (e.g., glacial-interglacial) during this MIS 8 interval, and with state changes in MIS 5 and 7.

Supplementary Note 3: Analytical methods and uncertainty

Individual specimens of the planktonic foraminifera *Globigerinoides sacculifer* without the final sac were analyzed for trace metals via laser ablation inductively coupled mass spectrometry (LA-ICPMS) following the protocol outlined by Sadekov et al. ([15]) and detailed by White et al. ([2]). Individuals were selected from the relatively narrow 355-425µm size fraction to reduce ontogenetic effects ([16]). In a departure from previous LA-ICPMS reconstructions that analyzed the final chamber from the inside out ([15], [4], [2]), we ablated from the outside of the test toward the inside (Supplementary Figure 8). Comparisons of outside-in and inside-out analyses on the f0 chamber showed that variability is similar between the two methods, but absolute Mg/Ca values are consistently offset by +0.2 mmol/mol (outside-in minus inside-out). This offset is consistent across measured Mg/Ca value (~2-5 mmol/mol), and may be the result of surface effects from the concave curved inner surface of the foraminifera. Our multiple-chamber method, however, allows us to calculate Mg/Ca ratios that include earlier ontogenetic signals from the f1 chamber and shows close correspondence with Mg/Ca ratios derived from traditional cleaning and dissolution methods.

Analytical and calibration uncertainty

Average intra-chamber standard deviation (1σ) of the three analyses of the f0 chamber is ±0.112 mmol/mol, and of the two analyses of the f1 chamber is ±0.107 mmol/mol. This translates to ±0.24 °C for f0 and ± 0.24 °C for f1 in a foraminifer with an average Mg/Ca ratio of 5 mmol/mol. Anomalies in ^{27}Al, ^{55}Mn, and ^{66}Zn were identified to indicate possible contamination from clays, metal hydroxides, or other sources. As such contamination was uncommon in these sediments, suspect chambers/individuals were removed from analysis. Trace element peaks associated with the inside and outside layers of foraminifera shells were automatically removed from the analysis via an automated function. NIST glass standard 610 was repeatedly analyzed during each analytical run to compute absolute response to, and drift in, elemental intensities during and across sample runs. NIST glass was analyzed at 4Hz and fluence between 0.59 and 0.76 J/cm^2 sufficient to obtain adequate signal strength and ablation duration for analysis. The estimated error for LA-ICP-MS Mg/Ca is 1.5%, based on the standard deviation of repeated measurements of NIST 610.

White et al. ([2]) estimated the total 1σ uncertainty in each foraminiferal Mg/Ca-derived temperature for site 14MC, based on analytical uncertainty (±0.225°C), salinity variations (±0.07°C), and dissolution (±0.3°C) in pore waters, to be ±0.4°C in quadrature. Our uncertainty includes analytical uncertainty (±0.24°C) for both f0 and f1 chambers and yields a total uncertainty of ±0.47°C. This uncertainty is effectively identical to that calculated in LA-ICPMS calibration studies using single specimens of *G. sacculifer* ([15]).

Impact of analytical uncertainty on ENSO change

To assess the impact of this analytical uncertainty of our determination of whether an interval shows changed El Niño amplitude, we applied bootstrap resampling to randomly altered temperature distributions. We generated random temperature uncertainties from a normal

distribution with mean zero and standard deviation of 0.47°C, and applied these random anomalies to each individual foraminifera temperature. We repeated this process 10^4 times and performed Q-Q analysis on each realization for each sample interval (**Supplementary Table 3**). For each realization, we determined whether each sample interval displayed changed El Niño amplitude, the number of significantly changed quantiles, and the effect on median absolute deviation. Intervals with increased El Niño amplitude in multiple quantiles are unlikely to change, intervals of only slightly enhanced El Niño amplitude (e.g., 1 quantile) may be influenced by uncertainties. Intervals with decreased El Niño amplitude are more sensitive to analytical uncertainty. While Interval MIS 7e is likely to display reduced El Niño amplitude with analytical uncertainty applied, but MIS 7a is highly sensitive to these results, and, as noted, the finding of reduced El Niño amplitude compared to the modern mixed layer is not robust. In most cases, analytical uncertainty increased the total variability of the population, although the median MAD of the Monte Carlo populations is within 1 standard deviation of the actual sample MAD.

Age and analytical uncertainty and correlations

We incorporate age and analytical uncertainty into our analysis of the relationship between MAD, the mean 94th-98th quantile temperature anomalies of our sample intervals and climate boundary conditions. We used weighted bivariate linear regression (WLR) routines ([17]) to determine correlations and statistical significance accounting for these uncertainties, and incorporated Monte Carlo techniques in order to estimate our uncertainties. First, we interpolate the value of each climate parameter at each of our interval ages. We estimate the uncertainty about this value by generating age uncertainties for each record, taking in to account the uncertainty of the specific climate record and the uncertainty of our age model in quadrature. We perform Monte Carlo simulations to generate multiple age models for each record by random alteration of the ages by an amount drawn from a normal distribution with the combined age

uncertainty. We then randomly applied analytical uncertainty for each record from a normal population with standard deviation of the analytical uncertainty, and then re-interpolated the data at the resulting age using the resulting analytical value. Total uncertainty about that value for use in the WLR algorithm is then calculated as one half of the ~2-sigma range. We estimate the 2-sigma range from our empirical Monte Carlo data using the 2.5 and 97.5 quantiles, which utilizes most of the generated data. Ages uncertainties for each record were taken from the original references when possible. Age and analytical uncertainty for insolation parameters was considered zero, as these parameters are mathematically solutions ([18]). Age uncertainty for the E-W SST gradient was estimated at 2ky, which is two times the time steps of the record. Analytical uncertainty in the original record was estimated at 0.5 °C. Age uncertainty for the latest portion of the ITCZ Ti MAR record was determined from published 14C dates ([19]). Ages for the remainder of the record used an alignment method similar to ours using Analyseries. We conservatively assigned this record a value of 2x our age errors. No analytical uncertainty was reported for this record, but we applied a 0.5% uncertainty factor based on the mean value of the Ti MAR data ([20]). The mixed layer $\delta^{18}O$ data from site 851 reported an analytical uncertainty of 0.07‰, which we added in quadrature to the mixed-layer calculation ([21]). We applied an age uncertainty of 3ky, which is the estimated uncertainty of the core used for alignment and age control ([22]). For the interpolated data points at 152ka and 162ka, we incorporate additional uncertainty about our interpolated data. We estimate this additional uncertainty from the amplitude of the dominant period of the Site 851 data (obliquity, ~41ky) during the early portion of the record ([21]). The maximum amplitude of such change is ~1.6‰ from 4-300ky. The maximum offset from our intervals to Site 851 data is less than 20% of one obliquity cycle. We therefore estimate that the data would likely be within 20% of this maximum amplitude, or

0.33‰, and apply this additional uncertainty to these data points. We estimate the uncertainty in our variability data from our foraminifera populations and apply this to our weighted bivariate regression as well. MAD uncertainty is calculated as the standard error of the MAD. For the 94-98th quantile mean, we our uncertainty as one half of the 2.5 and 97.5 quantile range, which approximates one standard deviation from our empirical data as above. We combine this with our calculated analytical uncertainty, in quadrature, to determine our total uncertainty. Climate parameter data and uncertainties are shown in **Supplementary Table 4**.

Supplementary Figures

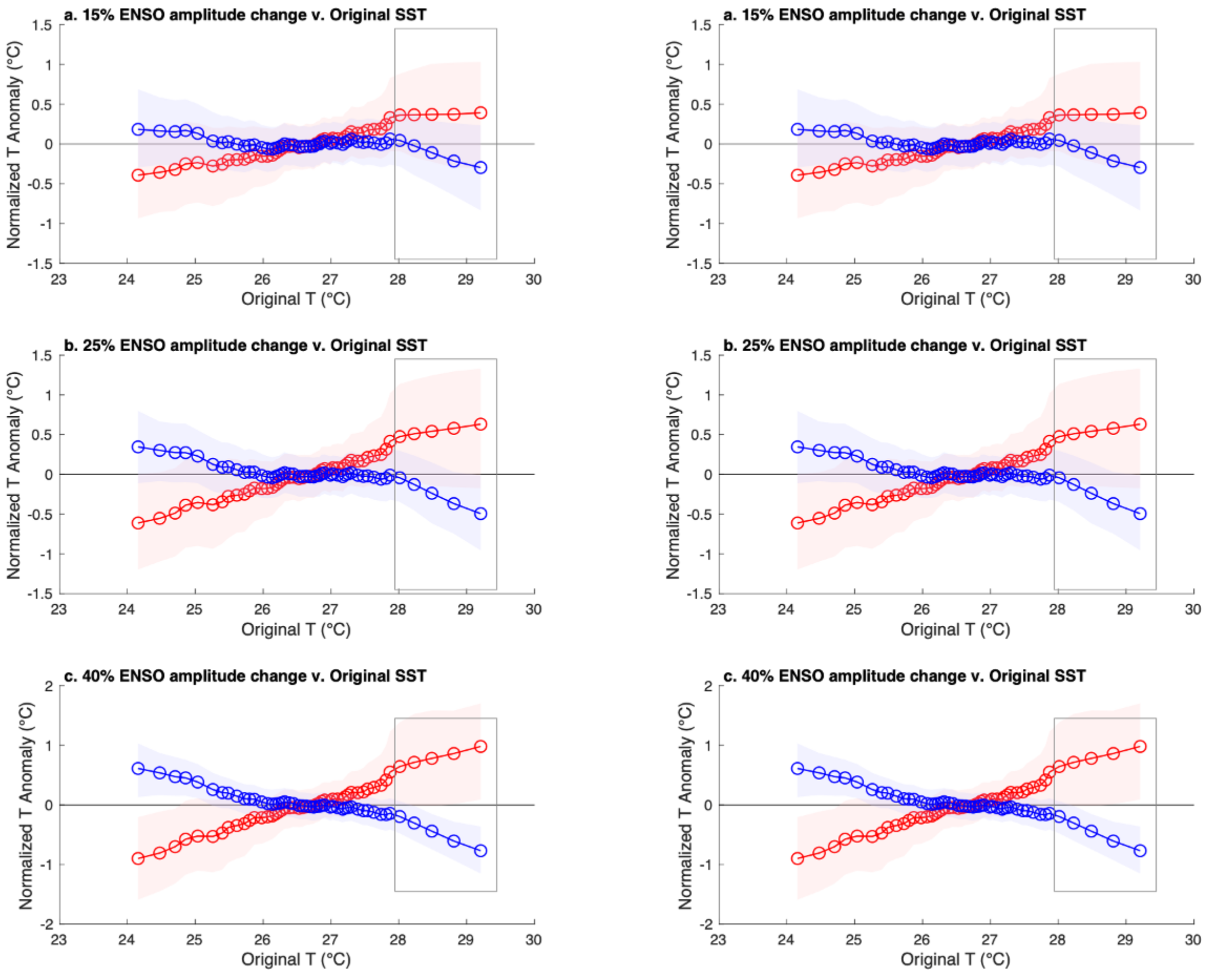

Supplementary Figure 1. Quantile-quantile comparisons of randomly resampled altered sea surface temperature distributions. Synthetic sea surface temperature (SST) records were generated by altering ENSO amplitude by 15%, 25% and 40%. Red lines denote results from increases in amplitude, blue lines denote decreases. Solid lines and open circles are the mean results of Monte Carlo simulations. Shaded regions are the 90% confidence intervals. a, b, and c show the results of randomly resampling 80 monthly SST values (foraminifera) from a 50-year time series. d, e, and f show the results when resampling from a 500-year time series, generated

by repeating the initial time series before alteration. The reference time series is the Carton 2.1.6 mixed-layer temperature data set from 1958-2008. Amplitude reduction of 25% will be identified in over 90% of cases. Amplitude increase of 50% will likewise be identified in over 90% of cases.

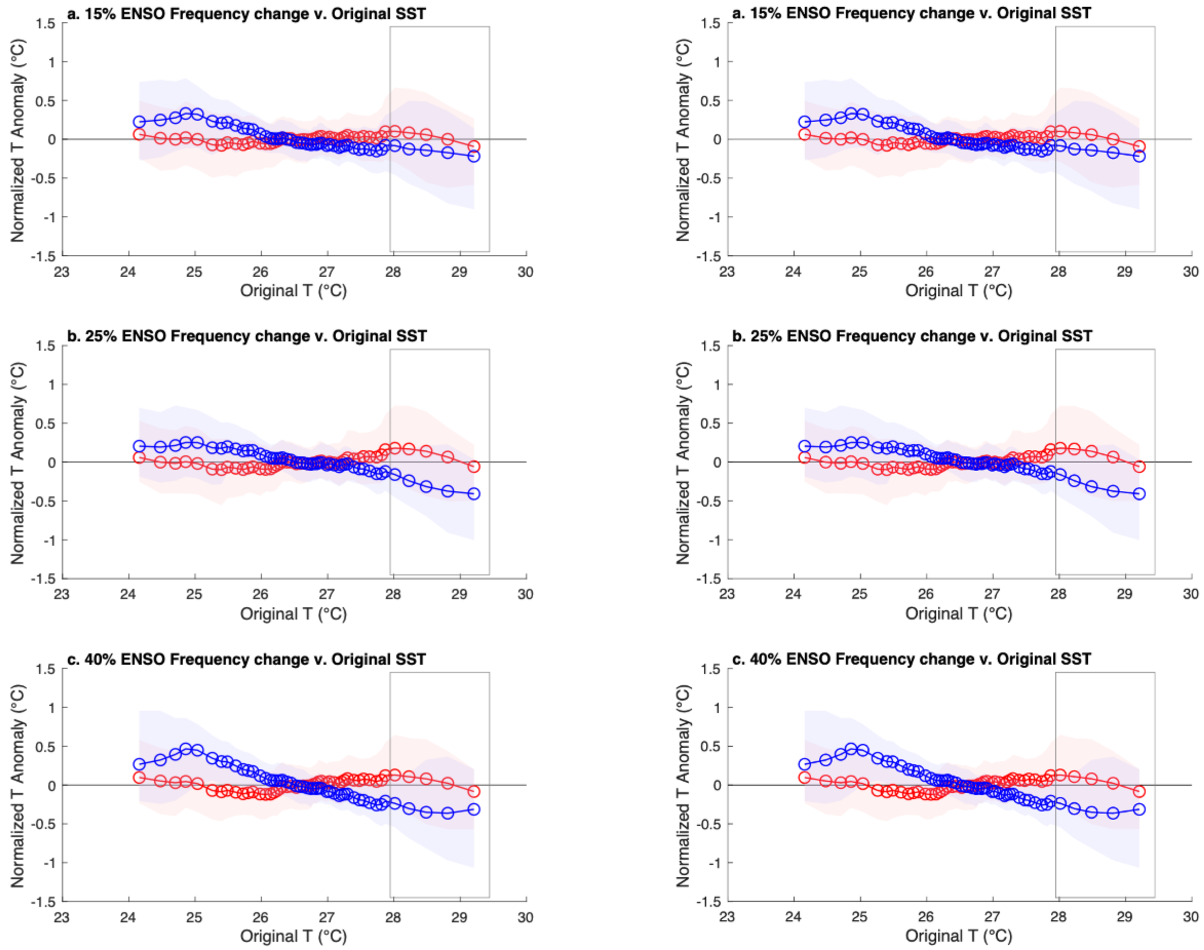

Supplementary Figure 2. Quantile-quantile comparisons of randomly resampled altered sea surface temperature distributions. Synthetic sea surface temperature (SST) records were generated by altering ENSO frequency by 15%, 25% and 40%. Red lines denote results from increases in frequency, blue lines denote decreases. Solid lines and open circles are the mean

results of Monte Carlo simulations. Shaded regions are the 90% confidence intervals. **a**, **b**, and **c** show the results of randomly resampling 80 monthly SST values (foraminifera) from a 50-year time series. d, **e,** and f show the results when resampling from a 500-year time series, generated by repeating the initial time series before alteration. The reference time series is the Carton 2.1.6 mixed-layer temperature data set from 1958-2008. Frequency reductions of up to 50% were unlikely to result in 90% likelihood of identification of such changes using individual foraminifera sampling.

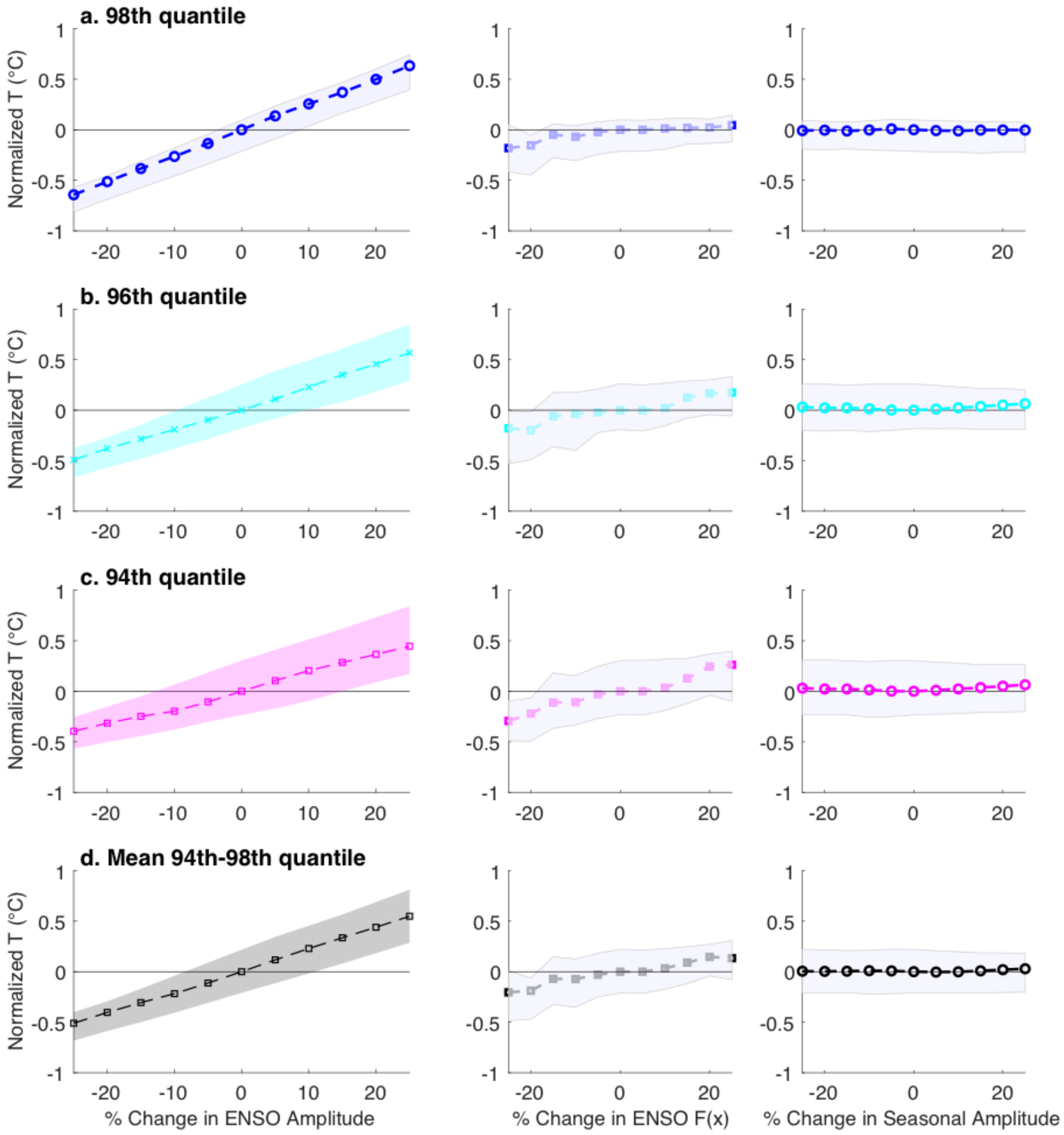

Supplementary Figure 3. Response of the normalized temperature of the 94th, 96th and 98th quantile and the 94th-96th quantile mean to changes in ENSO parameters. All simulations use the same reanalysis data as above in Supplementary Figures 1 and 2. Vertical scale is normalized temperature for all plots. Left column shows the response to change in ENSO amplitude. Middle column shows response to change in ENSO frequency, which were simulated

10X to account for randomness in the addition/deletion of ENSO events. The right column shows the response to changes in the seasonal cycle. Shaded region for a-c shows the 90% confidence interval based on bootstrap resampling. a) Response of the 98th quantile to changes in ENSO parameters. b) Response of the 96th quantile temperature to changing ENSO parameters. c) Response of the 94th quantile temperatures changes in ENSO parameters. d) Response of the mean of the 94th-98th quantiles. Shaded region is the estimated 1-sigma uncertainty, as calculated from bootstrap resampling. In all simulations, changes in seasonality have little effect. Changes in frequency have small, but not statistically significant effects, at the 98th and 96th quantiles.

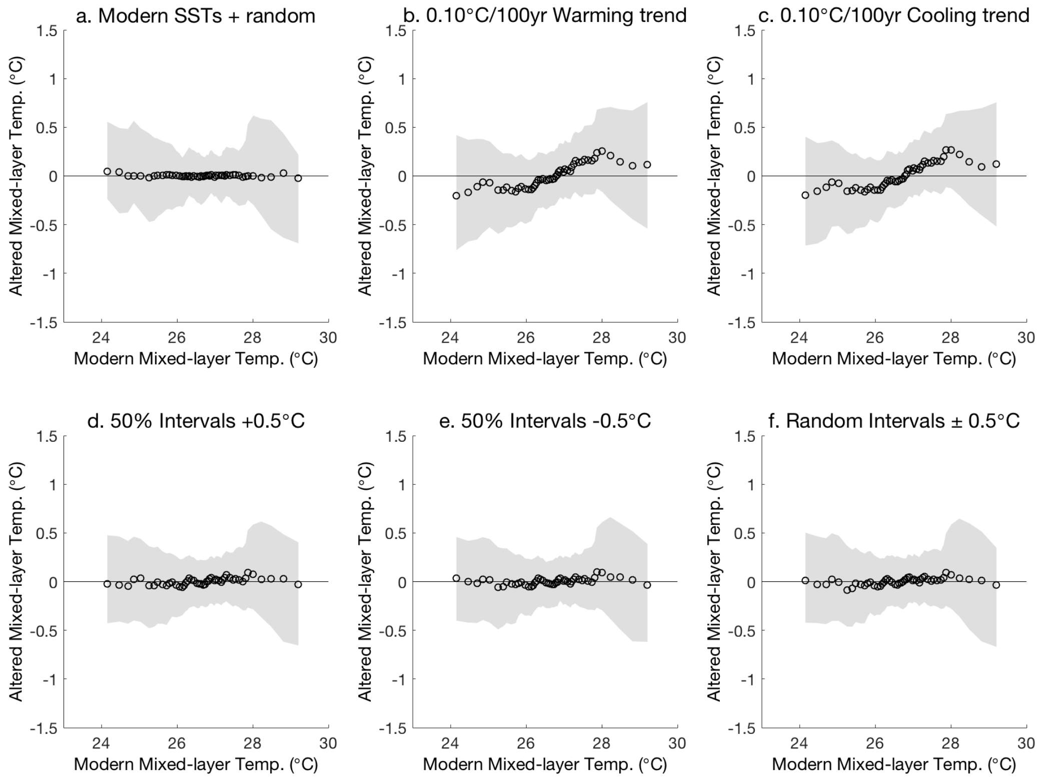

Supplementary Figure 4. Normalized quantile-quantile plots showing the effect of centennial-scale variability on mixed-layer temperature distributions. The common

reference interval for all simulations is the Carton 2.1.6 35m temperature for the study location from 1958-2009 ([23]). a) Normalized Q-Q for a generated 2000-year time series based on appending the original time series and adding random noise (median absolute deviation (MAD) = 0.71); b) Normalized Q-Q for a generated 2500-year time series, as before, with a 0.1°C/century warming trend added in addition to random noise (MAD = 0.85); c) Same as b, but with a 0.5°C cooling trend (MAD = 0.85); d) Same as a, but with 50% of appended intervals having a mean temperature +0.5°C higher than the initial data set (MAD = 0.74); e) Same as d, but with a -0.5°C mean temperature (MAD = 0.74); f) Normalized Q-Q for a generated 2500-year time series with random 50-year intervals with mean temperatures that are unchanged, +0.5°C or -0.5°C (MAD = 0.75).

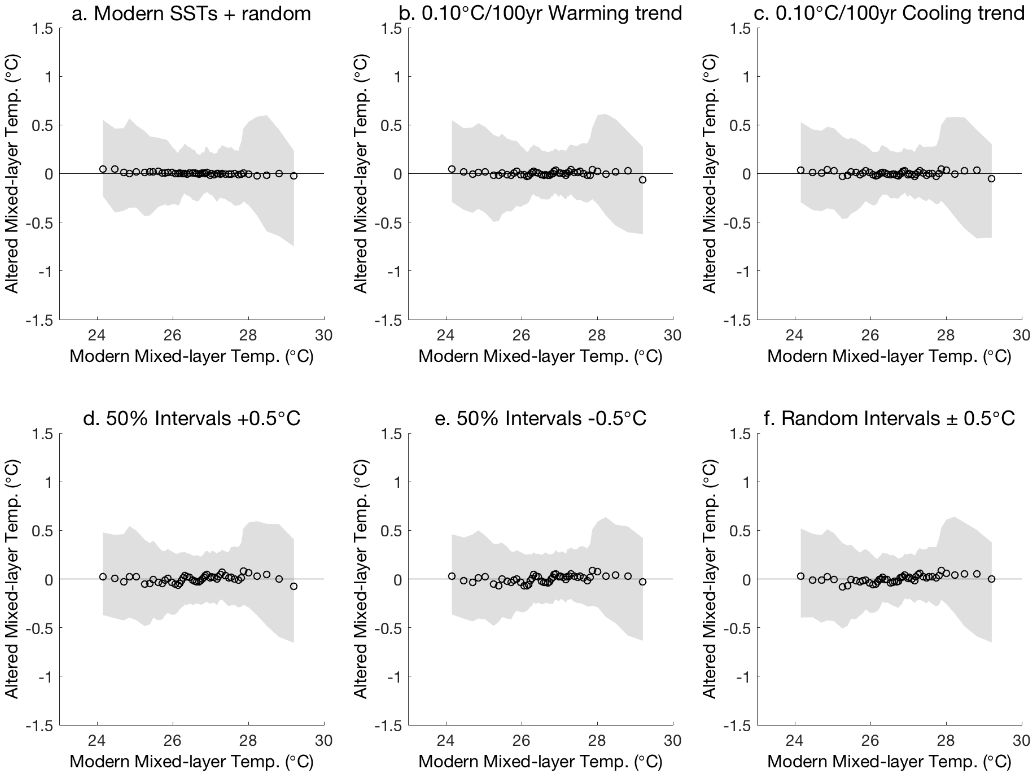

Supplementary Figure 5. Normalized quantile-quantile plots showing the effect of centennial-scale variability on mixed-layer temperature distributions. The common reference interval for all simulations is the Carton 2.1.6 35m temperature for the study location from 1958-2009 ([23]). a) Normalized Q-Q for a generated 400-year time series based on appending the original time series and adding random noise (median absolute deviation (MAD) = 0.71); b) Normalized Q-Q for a generated 400-year time series, as before, with a 0.1°C/century warming trend added in addition to random noise (MAD = 0.72); c) Same as b, but with a 0.5°C cooling trend (MAD = 0.72); d) Same as a, but with 50% of appended intervals having a mean temperature +0.5°C higher than the initial data set (MAD = 0.74); E) Same as d, but with a -0.5°C mean temperature (MAD = 0.74); f) Normalized Q-Q for a generated 400-year time series

with random 50-year intervals with mean temperatures that are unchanged, +0.5°C or -0.5°C (MAD = 0.74).

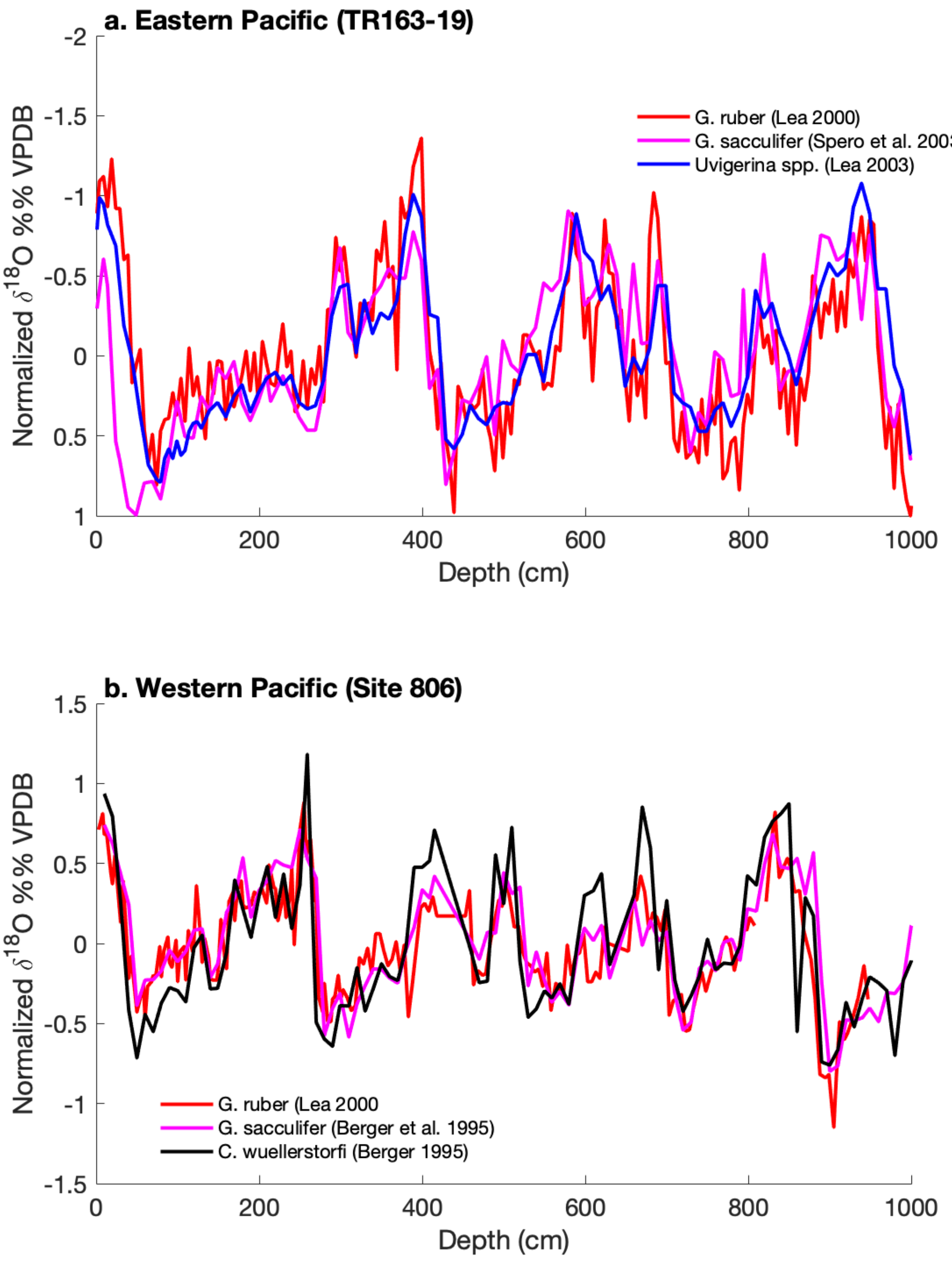

Supplementary Figure 6. Oxygen isotope records showing benthic-planktic $\delta^{18}O$ age offsets. Normalized oxygen isotope stratigraphies from the a. Eastern Tropical Pacific warm pool

(site TR163-19, 2.25°N, 90.95°W) ([11],[12]) and b. Western Tropical Pacific (site 806, 0.19 °N, 159.22°E) ([10],[11]). Each record was normalized to the record average over the time period analyzed. Surface-dwelling species (*Globigerinoides ruber, Globigerinoides sacculifer*) show no significant offsets from benthic species (*Uvigerina spp.* or *Cibicidoides wuellerstorfi*) at major glacial/interglacial transitions. Such transitions are characterized by large vertical shifts in each record.

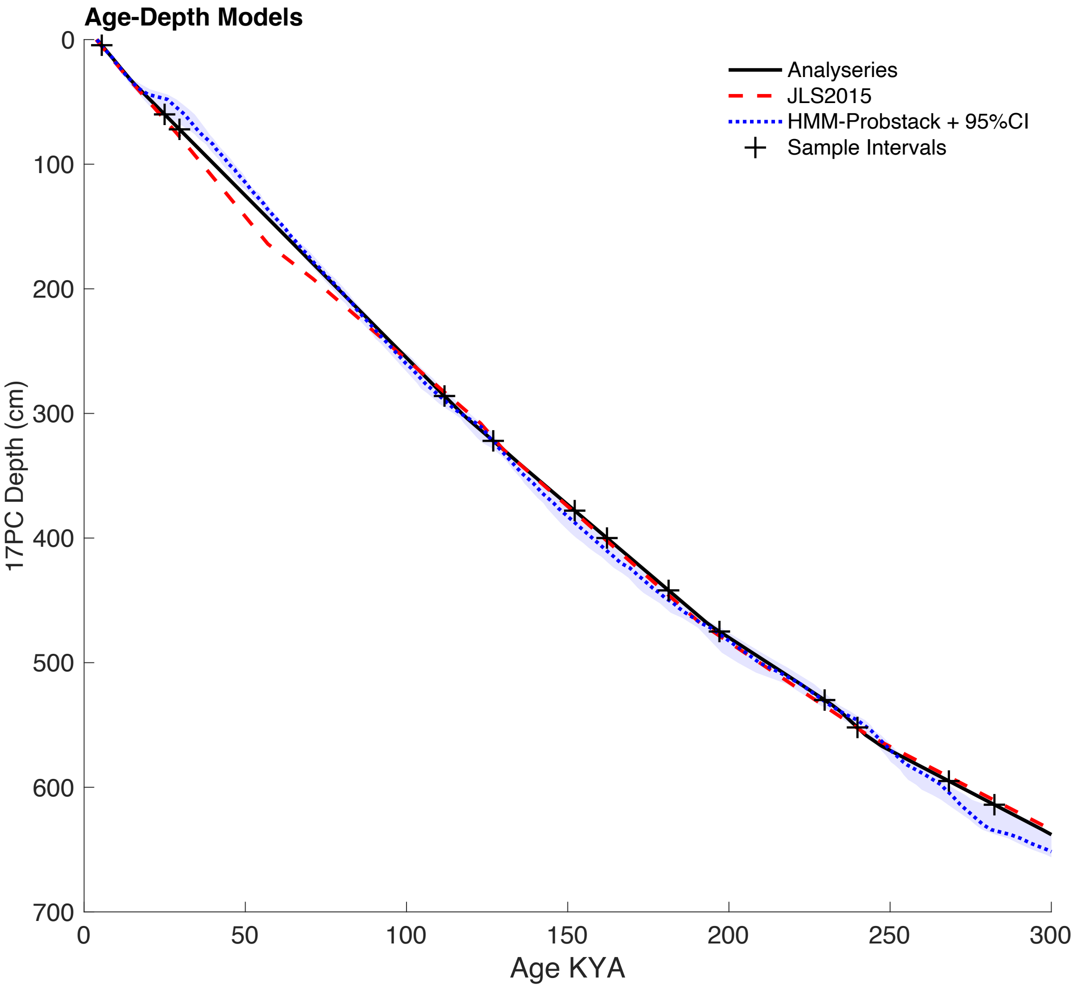

Supplementary Figure 7. Age-depth models for core ML1208-17PC. Lynch-Steiglitz et al. ([7]) ("JLS", red dashed line) is based on alignment with the LR04 benthic stack at isotope stage

transitions. HMM-Probstack based age model is generated via Bayesian age modeling ([14]) (blue dotted line). Our age model is based on alignment with the LR04 stack using repeated Analyseries alignments.

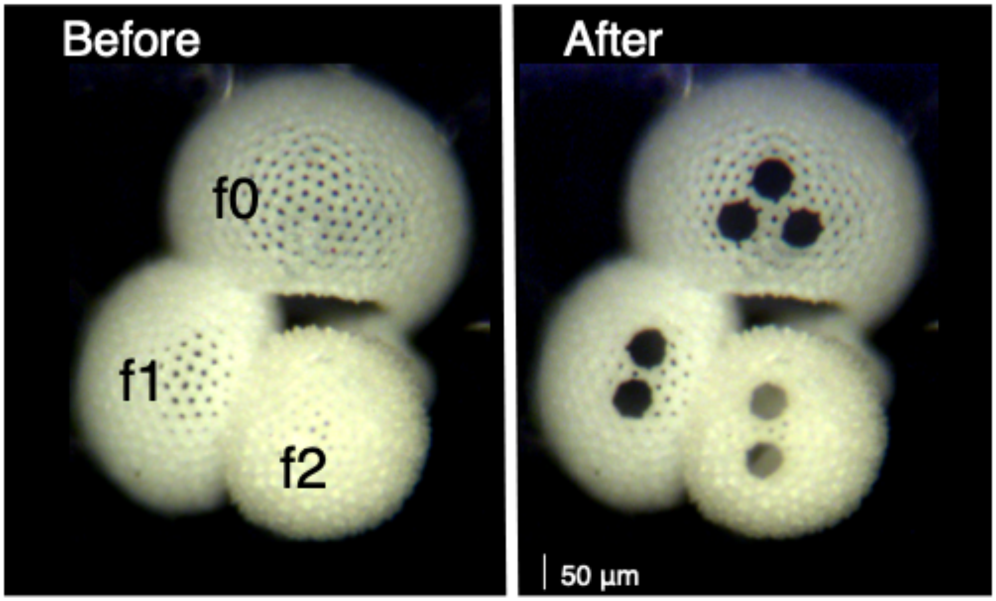

Supplementary Figure 8. Specimen of *G. sacculifer* before and after laser ablation. Ablation targets for these holes was a 50μm target. Each hole ablates for 20-300 seconds to completely burn through. Multiple sites were targeted for ablation on each chamber, and multiple chambers were targeted for each individual foraminifer. Mg/Ca values from targets on chambers f0 and f1 were used to calculate Mg/Ca representative of the whole foraminifer.

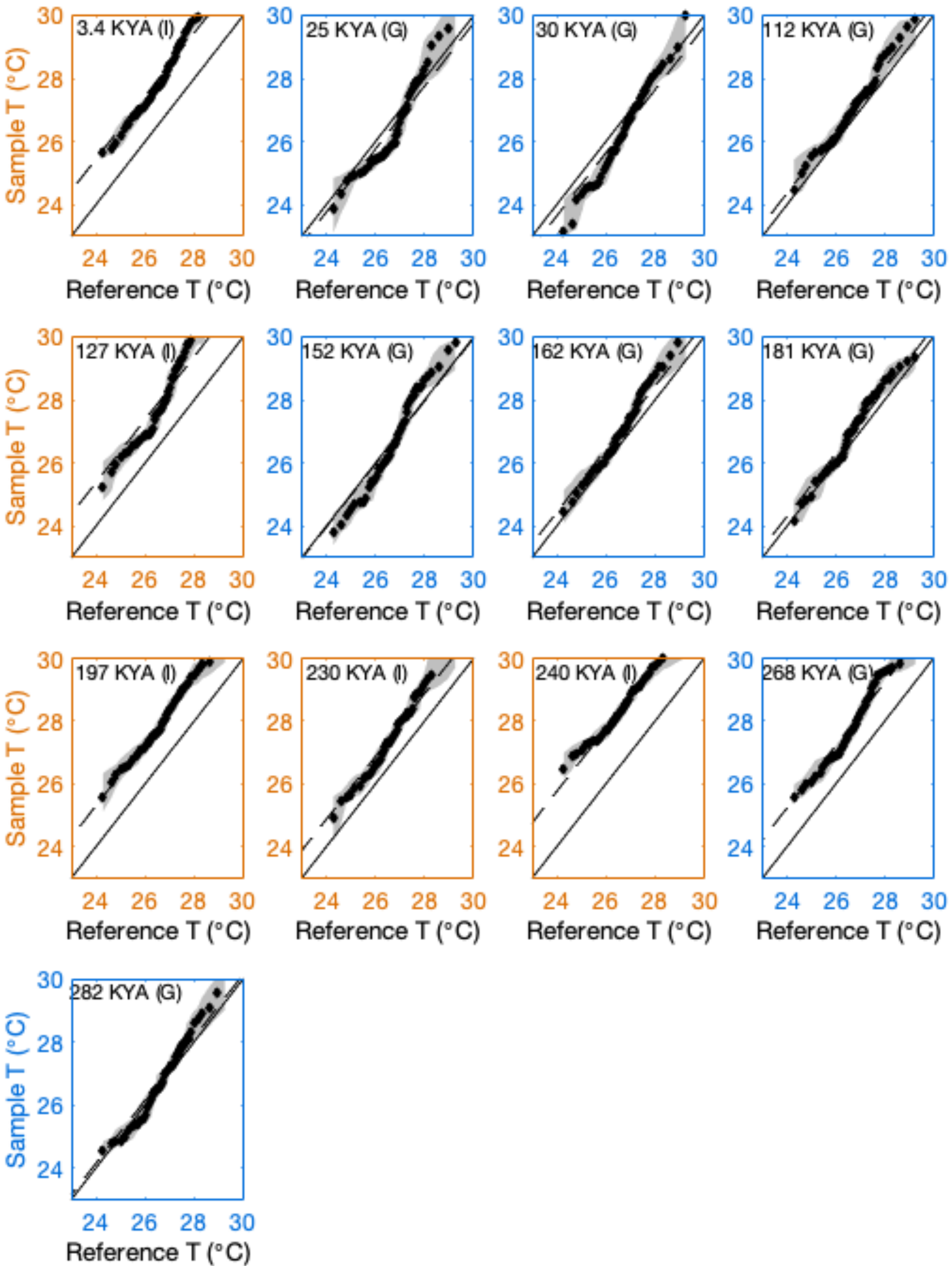

Supplementary Figure 9. Quantile-Quantile plots for sample intervals. Interglacial intervals are outlined in orange and marked with (I), glacial intervals are outlined in blue and marked with

(G). The one-to-one (1:1) line is the solid diagonal line. The dotted line above or below the 1:1 line runs through the mean of the sample intervals with slope 1 to show temperature offset. The reference interval for all intervals is modern mixed-layer temperatures (1958-2009, 15-46m) from the 0.5°x0.5° cell surrounding the site of 17PC in the Central Equatorial Pacific ([23]).

Supplementary Tables

Supplementary Table 1.**Interval depths and ages from 14MC and 17PC.** Ages from our age model are shown in column two, ages from previous studies in columns 3-4. 17PC ages for this study were generated from alignment with the benthic LR04 ([8]) stack using Analyseries and nine tie points as well as published radiocarbon dates from the core top. Ages from Lynch-Stieglitz were from alignment with the LR04 stack using seven age control points ([7]). Ages from Jacobel et al. ([13]) using MonteXCM for sediments representing the past 150ky. Single asterisks (*) indicate core-top radiocarbon dates from intervals not analyzed in this study but used for age modeling. Double asterisks (**) denote radiocarbon data for sediments from core 14MC1 ([2]). X's denote intervals not dated in the corresponding study.

**Core ID**	**Core depth (cm)**	**This study (ka)**	**Lynch-Stieglitz et al. 2015 (ka)**	**Jacobel et al. 2017 (ka)**
ML1208-14MC1	0**	4.0**	x	X
ML1208-17PC	0*	4.03*	4.03*	4.97*
ML1208-14MC1	4.5**	3.4**	x	x
ML1208-17PC	60	24.97	23.55	24.31
ML1208-17PC	72	29.58	27.64	30.08
ML1208-17PC	286	111.79	113.13	109.7
ML1208-17PC	322	126.9	127.67	127.26
ML1208-17PC	378	152.17	151.51	x
ML1208-17PC	400	162.18	161.16	x
ML1208-17PC	442	181.29	179.59	x
ML1208-17PC	475	197.06	195.04	x
ML1208-17PC	530	229.69	226.82	x
ML1208-17PC	552	239.87	239.54	x
ML1208-17PC	595	268.23	270.75	x
ML1208-17PC	614	282.35	285	x

Supplementary Table 2. Age control points for core 17PC for Analyseries alignment.

Depths are the depth in centimeters of the age control point selected on the 17PC $\delta^{18}O$ stratigraphy. Age is in ky, tuned to the LR04 ([8]) benthic $\delta^{18}O$ stack.

Iteration 1		Iteration 2		Iteration 3		Iteration 4		Iteration 5	
**Depth**	**Age**	**Depth**	**Age**	**Depth**	**Age**	**Depth**	**Age**	**Depth**	**Age**
34	15.4	34.2	15.4	33.6	15.2	32.1	14.3	33.2	14.4
300.9	116.7	300.6	118.2	303.4	118.7	302.2	116	302.5	118.5
336.9	133.2	337.4	133.7	336.5	133.6	331.4	133.1	336.9	133.3
469.6	193.7	469.3	193.5	470.2	193.8	469.5	193.7	469.9	193.6
536.3	232.7	536.1	233.7	536.8	234.2	535.8	233.7	536.3	233.2
564.4	244.3	564.5	244.5	562.8	244.4	565.4	244.3	562.8	244.4
652.5	309.5	652.5	310.3	652.1	311.2	653.2	310	653.2	309.7
700.8	338.4	701	339.1	701.6	339	701.3	338.7	701.2	338.2
765.2	380.5	767.7	383	769.4	382	769	384.1	769.4	382.5
**Iteration 6**		**Iteration 7**		**Iteration 8**		**Iteration 9**		**Iteration 10**	
**Depth**	**Age**	**Depth**	**Age**	**Depth**	**Age**	**Depth**	**Age**	**Depth**	**Age**
35	15	32.9	15.5	32.7	13.4	34.8	14.8	33.9	15.1
301.7	118.3	301.9	118.4	301.4	116.7	303.5	118.5	299.9	118.4
336.5	133.2	336.9	133.7	337.7	133.6	338.3	133.8	340.8	133.8
470.1	193.8	468.9	193.9	470	194	469.6	194.9	469.9	194.1
536.8	232.6	537.9	234.2	536.8	234	535.8	234.4	512.8	220.6
564.4	244.4	561.2	244.5	561.7	244.3	560.1	243.7	560.6	244.2
653.2	310.1	651.6	309	652.3	309.5	634.3	301.4	634.6	301.2
701.7	338	701.1	338.4	701.4	338.1	700.5	338	700.6	338.9
768.3	383	768.3	382	768.6	382.3	767.3	382.9	769	382.9

Supplementary Table 3. Results from Monte Carlo simulation of analytical uncertainty.

For each interval, each individual foraminifera temperature was altered by an amount randomly drawn from a normal distribution with standard deviation of 0.47 °C. Q-Q analysis were performed using the reference interval, and MAD was calculated. The %+ indicates the percentage of realizations that report at least one quantile in the 90-98th region that is statistically increased, %- shows the percentage of realizations showing significant reduction in El Niño amplitude. The #Q sig column shows the number of quantiles in the 90-98th range that show

significant positive or negative El Niño amplitude change (negative numbers indicate negative change). Actual MAD is the MAD for the unchanged sample interval. Synth. MAD is the median MAD of all of the synthetic populations for an interval with additional uncertainty, and the standard deviation of the MAD for all realizations. Median MAD is elevated, compared to actual MAD, in all cases except at 162ky (MIS6) when additional uncertainty is added. Intervals with large numbers of quantiles that show significant positive or negative El Niño amplitude change show little change due to analytical uncertainty.

**Interval Age**	**MIS**	**% +**	**% -**	**#Q sig**	**Sample MAD**	**Synth. MAD**
3.4	Holocene	1.00	0	4	1.00 ±0.09	1.02 ±0.07
25.0	MIS2	0.91	0	1	0.96 ±0.16	1.04 ±0.10
29.6	MIS3	0.93	0	2	1.08 ±0.14	1.14 ±0.09
111.8	MIS5d	0.13	0.02	0	0.75 ±0.13	0.88 ±0.10
127.0	MIS5e	1.00	0	4	1.14 ±0.15	1.20 ±0.09
152.2	MIS6	1.00	0	3	1.21 ±0.15	1.25 ±0.10
162.2	MIS6	0.73	0	0	1.08 ±0.13	1.04 ±0.09
181.3	MIS6	0.41	0.07	0	1.00 ±0.12	1.04 ±0.09
197.1	MIS7a	0.09	0.28	-1	0.83 ±0.11	0.90 ±0.08
229.7	MIS7d	0.42	0	0	0.93 ±0.13	0.99 ±0.09
239.9	MIS7e	0.00	0.67	-4	0.74 ±0.10	0.81 ±0.08
268.2	MIS8	0.60	0	1	0.97 ±0.12	1.03 ±0.08
282.4	MIS8	0.51	0	0	0.96 ±0.13	1.03 ±0.09

Supplementary Table 4. Data and uncertainty estimates for data and ages. Ages and 1-sigma age uncertainty from our Age Model using Analyseries and $\delta^{18}O$ stratigraphy from 17PC ([7]). Total uncertainty for variability estimates and climate parameters is calculated as one half of the ~2-sigma empirical range from Monte Carlo resampling and the analytical uncertainty in quadrature unless noted. 94_98T is the mean of the normalized 94th-98th quantile temperature anomalies. Median absolute deviation (MAD) is calculated from the reconstructed SST data from the CEP, MAD uncertainty is the standard error of MAD. Insolation parameters are for 0° June insolation ([18]), uncertainty is calculated via Monte Carlo resampling using the age uncertainty of the 17PC age model. The east-west sea surface temperature (EW SST) gradient had a 2ky age uncertainty and 0.5°C analytical uncertainty applied ([24]). Ti MAR uncertainties are estimates as twice the age uncertainty of 17PC with the exception of the first three intervals where published ^{14}C uncertainties are used ([19]). Mass accumulation rate (MAR) uncertainty estimated at 5% of the mean MAR ([20]). Mixed-layer $\delta^{18}O$ is the difference between *G. tumida* and *G. sacculifer* at site 851 in the eastern tropical Pacific. Age uncertainty is estimated at 3ky ([22]), and analytical uncertainty is reported at 0.07‰ for each foraminifera record, in quadrature ([21]).

**Age (ky)**	**Age 1-sig unc.**	**94_98T**	**94_98T unc.**	**MAD**	**MAD unc.**	**Insolation (W/m2)**	**Ins. unc**	**EW Gradient (°C)**	**EW unc.**	**Ti MAR (gmcm/kgyr)**	**Ti MAR unc.**	**Mixed-layer contrast ($\delta^{18}O$)**	**MLC unc**
3.4	0.2	0.56	0.44	1	0.09	390	0.3	6.09	0.42	1.09	0.06	1.87	0.10
25	0.5	1.05	0.68	0.96	0.16	393.2	1.1	6.36	0.5	0.39	0.05	1.12	0.21
29.6	0.5	0.62	0.55	1.08	0.14	408.3	1.9	6.29	0.42	0.34	0.04	1.33	0.20
111.8	0.9	0.31	0.51	0.75	0.13	403.3	8	5.48	0.42	0.82	0.08	1.71	0.17
126.9	0.4	0.75	0.52	1.14	0.15	433.3	0.6	5	0.58	0.73	0.06	1.49	0.14
152.2	0.6	0.57	0.49	1.21	0.15	427.3	0.9	6	0.42	0.44	0.06	1.49	0.45
162.2	0.5	0.41	0.52	1.08	0.13	383.3	1.4	4.8	0.46	0.41	0.06	1.67	0.44
181.3	0.4	-0.02	0.42	1	0.12	408.3	2.6	4.15	0.44	0.68	0.06	1.68	0.20
197.1	0.4	-0.16	0.41	0.83	0.11	440.1	2	6.24	0.48	1.24	0.08	2.09	0.15
229.7	0.7	0.43	0.53	0.93	0.13	380.2	3.5	4.76	0.43	1.79	0.17	1.48	0.32

239.9	0.5	-0.54	0.38	0.74	0.1	427.1	3.1	5.33	0.61	1.12	0.11	2.58	0.36
268.2	1.5	0.1	0.46	0.97	0.12	421	1.4	6.79	0.46	0.83	0.05	1.38	0.20
282.4	1.8	0.48	0.53	0.96	0.13	386.1	3.9	6.34	0.48	0.74	0.09	1.92	0.18

Supplementary References

1. Rustic, G. T., Koutavas, A., Marchitto, T. M. & Linsley, B. K. Dynamical excitation of the tropical Pacific Ocean and ENSO variability by Little Ice Age cooling. *Science* aac9937 (2015). doi:10.1126/science.aac9937
2. White, S. M., Ravelo, A. C. & Polissar, P. J. Dampened El Niño in the Early and Mid-Holocene Due To Insolation-Forced Warming/Deepening of the Thermocline. *Geophys. Res. Lett.* **45**, 316–326 (2017). doi:10.1002/2017GL075433
3. Koutavas, A. & Joanides, S. El Niño–Southern Oscillation extrema in the Holocene and Last Glacial Maximum. *Paleoceanography* **27**, PA4208 (2012). doi:10.1029/2012PA002378
4. Ford, H. L., Ravelo, A. C. & Polissar, P. J. Reduced El Niño–Southern Oscillation during the Last Glacial Maximum. *Science* **347**, 255–258 (2015). doi:10.1126/science.1258437
5. Thirumalai, K., Partin, J. W., Jackson, C. S. & Quinn, T. M. Statistical constraints on El Niño Southern Oscillation reconstructions using individual foraminifera: A sensitivity analysis. *Paleoceanography* **28**, 401–412 (2013). doi:10.1002/palo.20037
6. Rongstad, B. L., Marchitto, T. M., Marks, G. S., Koutavas, A., Mekik, F. & Ravelo, A. C. Investigating ENSO-Related Temperature Variability in Equatorial Pacific Core-Tops Using Mg/Ca in Individual Planktic Foraminifera. *Paleoceanogr. Paleoclimatology* **35**, e2019PA003774 (2020). doi:10.1029/2019PA003774
7. Lynch-Stieglitz, J., Polissar, P. J., Jacobel, A. W., Hovan, S. A., Pockalny, R. A., Lyle, M., Murray, R. W., Ravelo, A. C., Bova, S. C., Dunlea, A. G., Ford, H. L., Hertzberg, J. E., Wertman, C. A., Maloney, A. E., Shackford, J. K., Wejnert, K. & Xie, R. C. Glacial-interglacial changes in central tropical Pacific surface seawater property gradients. *Paleoceanography* **30**, 423–438 (2015). doi:10.1002/2014PA002746

8. Lisiecki, L. E. & Raymo, M. E. A Pliocene-Pleistocene stack of 57 globally distributed benthic δ18O records. *Paleoceanography* **20**, (2005). doi:10.1029/2004PA001071

9. Paillard, D., Labeyrie, L. D. & Yiou, P. AnalySeries 1.0: a Macintosh software for the analysis of geophysical time-series. *Eos* **77**, (1996).

10. Berger, W. H. IGBP PAGES/World Data Center-A for Paleoclimatology Data Contribution Series # 96-006. (1996).

11. Lea, D. W., Pak, D. K. & Spero, H. J. Climate Impact of Late Quaternary Equatorial Pacific Sea Surface Temperature Variations. *Science* **289**, 1719–1724 (2000). doi:10.1126/science.289.5485.1719

12. Spero, H. J., Mielke, K. M., Kalve, E. M., Lea, D. W. & Pak, D. K. Multispecies approach to reconstructing eastern equatorial Pacific thermocline hydrography during the past 360 kyr. *Paleoceanography* **18**, (2003). doi:10.1029/2002PA000814

13. Jacobel, A. W., McManus, J. F., Anderson, R. F. & Winckler, G. Climate-related response of dust flux to the central equatorial Pacific over the past 150 kyr. *Earth Planet. Sci. Lett.* **457**, 160–172 (2017). doi:10.1016/j.epsl.2016.09.042

14. Ahn, S. A probabilistic Pliocene-Pleistocene stack of benthic *δ^{18}O* using a profile hidden Markov model. *J. Clim.* **1**, (2017).

15. Sadekov, A., Eggins, S. M., Deckker, P. D., Ninnemann, U., Kuhnt, W. & Bassinot, F. Surface and subsurface seawater temperature reconstruction using Mg/Ca microanalysis of planktonic foraminifera Globigerinoides ruber, Globigerinoides sacculifer, and Pulleniatina obliquiloculata. *Paleoceanography* **24**, (2009). doi:10.1029/2008PA001664

16. Elderfield, H., Vautravers, M. & Cooper, M. The relationship between shell size and Mg/Ca, Sr/Ca, δ18O, and δ13C of species of planktonic foraminifera. *Geochem. Geophys. Geosystems* **3**, 1–13 (2002). doi:10.1029/2001GC000194

17. Thirumalai, K., Singh, A. & Ramesh, R. A MATLAB™ code to perform weighted linear regression with (correlated or uncorrelated) errors in bivariate data. *J. Geol. Soc. India* **77**, 377–380 (2011). doi:10.1007/s12594-011-0044-1

18. Laskar, J., Fienga, A., Gastineau, M. & Manche, H. La2010: a new orbital solution for the long-term motion of the Earth. *Astron. Astrophys.* **532**, A89 (2011).

19. Leduc, G., Vidal, L., Tachikawa, K., Rostek, F., Sonzogni, C., Beaufort, L. & Bard, E. Moisture transport across Central America as a positive feedback on abrupt climatic changes. *Nature* **445**, 908–911 (2007). doi:10.1038/nature05578

20. Rincón-Martínez, D., Lamy, F., Contreras, S., Leduc, G., Bard, E., Saukel, C., Blanz, T., Mackensen, A. & Tiedemann, R. More humid interglacials in Ecuador during the past 500 kyr linked to latitudinal shifts of the equatorial front and the Intertropical Convergence Zone in the eastern tropical Pacific. *Paleoceanogr. Paleoclimatology* **25**, (2010). doi:10.1029/2009PA001868

21. Cannariato, K. G. & Ravelo, A. C. Pliocene-Pleistocene evolution of eastern tropical Pacific surface water circulation and thermocline depth. *Paleoceanography* **12**, 805–820 (1997). doi:10.1029/97PA02514

22. Imbrie, J., Hays, J. D., Martinson, D. G., McIntyre, A., Mix, A. C., Morley, J. J., Pisias, N. G., Prell, W. L. & Shackleton, N. J. The orbital theory of Pleistocene climate: support from a revised chronology of the marine d18O record. (1984).

23. Carton, J. A. & Giese, B. S. A Reanalysis of Ocean Climate Using Simple Ocean Data Assimilation (SODA). *Mon. Weather Rev.* **136**, 2999–3017 (2008). doi:10.1175/2007MWR1978.1

24. Dyez, K. A. & Ravelo, A. C. Late Pleistocene tropical Pacific temperature sensitivity to radiative greenhouse gas forcing. *Geology* **41**, 23–26 (2013). doi:10.1130/G33425.1
